# The *C*. *elegans* truncated insulin receptor DAF-2B regulates survival of L1 arrested larvae

**DOI:** 10.1371/journal.pone.0288764

**Published:** 2023-07-20

**Authors:** Bryan A. Martinez, Matthew S. Gill

**Affiliations:** Institute on the Biology of Aging and Metabolism and the Department of Genetics, Cell Biology and Development, University of Minnesota, Minneapolis, MN, United States of America; Pontificia Universidad Catolica de Chile, CHILE

## Abstract

We have previously characterized a truncated isoform of the *C*. *elegans* insulin-like receptor, DAF-2B, which retains the ligand binding domain but cannot transduce a signal due to the absence of the intracellular signaling domain. DAF-2B modifies insulin / insulin-like growth factor signaling-dependent processes, such as dauer formation and lifespan, by sequestering insulin-like peptides (ILP) and preventing signaling through full length DAF-2 receptors. Here we show that DAF-2B is also important for starvation resistance, as genetic loss of *daf-2b* reduces survival in arrested first stage larvae (L1). Under fed conditions, we observe *daf-2b* splicing capacity in both the intestine and the hypodermis, but in starved L1s this becomes predominantly hypodermal. Using a novel splicing reporter system, we observe an increase in the ratio of truncated to full length insulin receptor splicing capacity in starved L1 larvae compared with fed, that may indicate a decrease in whole body insulin responsiveness. Consistent with this, overexpression of DAF-2B from the hypodermis, but not the intestine, confers increased survival to L1 animals under starvation conditions. Our findings demonstrate that the truncated insulin receptor DAF-2B is involved in the response to L1 starvation and promotes survival when expressed from the hypodermis.

## Introduction

Nutrient deprivation in juvenile *C*. *elegans* imperils their ability to grow and reproduce, which prompts animals to adopt metabolic and developmental strategies to maximize survival [1]. One well studied developmental pathway in *C*. *elegans* is the dauer diapause, a specialized alternate third larval stage that is selected when a combination of environmental conditions are poorly suited for reproduction. In addition, if animals hatch from the egg into a nutrient-depleted environment, they undergo arrest at the first larval stage (L1) instead. This L1 diapause is associated with cell division arrest and activation of stress resistance pathways but occurs without extensive morphological changes [2, 3].

The insulin / insulin-like growth factor signaling (IIS) pathway is indispensable for development, stress resistance and longevity in *C*. *elegans* [4–8] and is one of several signaling pathways that are altered during L1 arrest [1]. When nutrient levels are high, the receptor tyrosine kinase DAF-2 responds to extracellular insulin-like peptides by orchestrating a phosphorylation cascade via conserved intracellular kinases, ultimately leading to phosphorylation and cytoplasmic retention of the FOXO transcription factor DAF-16 [9]. In contrast, when nutrient levels are low, reduced insulin signaling leads to unphosphorylated DAF-16 entering the nucleus where it activates gene expression programs involved in stress resistance [10]. Correspondingly, hypomorphic mutations in *daf-2* that reduce insulin-signaling lead to increased survival during L1 starvation and *daf-16* loss-of-function mutations lead to premature death [11].

The *C*. *elegans* genome encodes forty insulin-like peptides (ILP) which differ in terms of their sequence similarity and predicted structure [12, 13], their spatial and temporal patterns of expression [14, 15], as well as their function [12, 16]. Interestingly, some of these ILPs act as agonists and others as antagonists of IIS, based on phenotypic effects of gene deletion or overexpression [12, 17–19]. In the context of L1 diapause, gene expression studies have indicated that starvation is associated with both downregulation of a subset of ILPs and upregulation of another subset [20]. This suggests that reduced insulin signaling during L1 arrest may arise from a reduction in ILP agonists, an increase in ILP antagonists or a combination of both. In support of the agonist model, it has been shown that combined deletion of *ins-4* and *daf-28* was sufficient to increase L1 survival [20]. In contrast, *ins-17*, one of the candidate antagonists that is upregulated in L1 arrest [20], was sufficient to promote L1 survival when overexpressed [19].

We have recently characterized DAF-2B, a secreted, truncated isoform of the DAF-2 insulin receptor that lacks the transmembrane and intracellular signaling domains but retains the ability to bind ILPs [21]. Our genetic studies suggest that DAF-2B acts as a modifier of insulin signaling through a decoy receptor / binding protein mechanism, whereby the protein sequesters insulin-like peptides and prevents their interaction with full length receptors. Interestingly, in the context of dauer formation, DAF-2B can promote or antagonize insulin signaling depending on the prevailing insulin environment. For instance, in IIS mutants that constitutively form dauers at high temperatures, the presence of DAF-2B promotes dauer entry and slows dauer recovery, consistent with sequestration of the agonist insulin-like peptides DAF-28 and INS-6 [21]. In contrast, in a pheromone paradigm of dauer formation, DAF-2B promotes insulin sensitivity by sequestering antagonist insulin peptides such as INS-18 [21].

Given the ability of DAF-2B to modify ILP-related phenotypes, we hypothesized that this truncated DAF-2 isoform may also play a role in L1 diapause. Here, we show that genetic loss of DAF-2B reduces L1 starvation survival, suggesting that its primary role in this context is to limit the activity of insulin agonists. Moreover, DAF-2B appears to function specifically in the hypodermis to antagonize insulin signaling and enhance L1 starvation survival. These data provide further evidence that DAF-2B acts as a modifier of IIS signaling and helps to provide plasticity to animals in adapting to constantly changing, uncertain environments.

## Methods

### Plasmids

All primers and plasmids are listed in S1 and S2 Tables in S1 File. To generate a *daf-2* dual splicing reporter, we first replaced the tdTomato sequence in pMGL83 (*daf-2a/c*::*tdTomato* splicing reporter) [21] with mNeon Green. mNeon Green was amplified from dg356 [22] with 5’ KpnI overhangs and 3’ *unc-54* 3’UTR overhangs (primers 1 & 2) and *unc-54* 3’UTR was amplified from pMGL86 using primers 3 & 4. The two fragments were fused by PCR using primers 1 & 4 and cloned into pMGL83 using KpnI and SpeI restriction digest to generate pMGL225 (*daf-2a/c*::*mNeon Green* splicing reporter). A 2.9kb fragment with 5’ XhoI and 3’ SpeI sites was amplified from pMGL225 using primers 5 and 4 and cloned into pMGL86 (*daf-2b*::*tdTomato* splicing reporter) cut with SalI and SpeI to generate pMGL226 (*daf-2b*::*tdTomato; daf-2a/c*::*mNeon Green* splicing reporter).

### *C*. *elegans* strains and maintenance

*C*. *elegans* strains were maintained as previously described [23]. Bristol N2 (wild-type), PD4667[*ayIs7[hlh-8*::*GFP* fusion + *dpy-20(+)*]], CF1038[*daf-16(mu86)*] were obtained from the *Caenorhabditis elegans* Genetics Center (University of Minnesota, MN). MGL264[*jluIs15[daf-2p*::*DAF-2bexon-11*.*5*::*tdTomato + rol-6(+)*]], MGL297[*jluSi3[daf-2p*::*DAF-2C + unc-119(+)*]], MGL302[*jluSi3[daf-2p*::*DAF-2C + unc-119(+)]; daf-2(jlu1)*]], MGL364[*jluIs15; jluEx180[dpy-7p*::*GFP*]], MGL365[*jluIs15; jluEx181[ges-1p*::*GFP*]], MGL370[*daf-2(jlu2[daf-2b*::*mScarlet]); jluEx184[unc-122p*::*GFP]* were previously reported [21]. All worm strains used in the study are listed in S3 Table in S1 File.

### Genetic crosses

Genetic crosses were performed using standard methods. MGL264[*jluIs15[daf-2p*::*DAF-2bexon-11*.*5*::*tdTomato + rol-6(+)*]] [21] was outcrossed twice to generate MGL371. We previously generated a *daf-2b* deletion (Δ) strain (MGL302) that carries a *daf-2bc* deletion and is rescued for *daf-2c* by a single copy insertion of *daf-2c* cDNA at the ttTi5605 Mos insertion locus [21]. The corresponding control strain MGL297 carries the *daf-2c cDNA* single copy insertion alone. CF1038 was outcrossed to the lab N2 3 times to generate MGL476. PD4667 and MGL476 were each crossed into MGL297 and MGL302 to generate MGL477[*daf-16(mu86); jluSi3[daf-2p*::*DAF-2C + unc-119(+)]*], MGL478[*daf-16(mu86); jluSi3[daf-2p*::*DAF-2C + unc-119(+)]; daf-2(jlu1)*], MGL474[*jluSi3[daf-2p*::*DAF-2C + unc-119(+)]; ayIs7[hlh-8*::*GFP fusion + dpy-20(+)]*], and MGL475[*jluSi3[daf-2p*::*DAF-2C + unc-119(+)]; daf-2(jlu1); ayIs7[hlh-8*::*GFP fusion + dpy-20(+)]*]. The *daf-2b(Δ)* locus was genotyped using primers 6–8, and the *daf-2c* Mos insertion was genotyped with primers 9–12. *Daf-16(mu86)* was genotyped with primers 13–15 and *hlh-8*::*GFP* homozygotes were identified by GFP.

### Transgenic strains

Strains overexpressing *daf-2b* cDNA in the hypodermis and the intestine were generated by injecting pMGL150(*tag-335p*::*daf-2b* cDNA) and pMGL123 (*ges-1p*::*daf-2b cDNA*) in N2 worms [21]. We used the *tag-335* promoter for hypodermal expression [24] as preliminary injections with the commonly used *dpy-*7 promoter did not yield transgenic lines, presumably due to the strength of the overexpression. Each construct was injected at a concentration of 25ng/μL with a *myo-2p*::*tdTomato* coinjection marker at a concentration of 5ng/μL. Three independent lines were analyzed for each construct in each background. A control line expressing only the *myo-2p*::*tdTomato* co-injection marker at 5ng/uL was generated by injection with 50ng/uL pBluescript vector.

A dual splicing reporter strain for *daf-2b*::tdTomato and *daf-2a/c*::mNeon Green was generated by injecting pMGL226 into N2 worms at a concentration of 50ng/uL with 50ng/ul *rol-6(+)* coinjection marker. A line stably expressing an extrachromosomal array was identified and the array was integrated with UV and outcrossed twice to create MGL456.

### L1 survival assay

The L1 survival assay was modified from Zhang et al. [25]. Eggs were obtained from approximately 2000 synchronous day 1 adults per condition by sodium hypochlorite treatment. Worms were washed from plates with sterile water and transferred to 15ml conical bottom centrifuge tubes. Worms were pelleted by gentle centrifugation and treated with hypochlorite solution (~1% sodium hypochlorite in 0.5M KOH) until adult carcasses started to dissolve. After pelleting and washing 2–3 times with sterile water, egg preparations were resuspended in S-basal (50mM KPO_4_ pH 6.0 and 100mM NaCl) without cholesterol. Tubes were placed on a rocking platform at 20°C overnight to hatch. After hatching, the volume was adjusted to yield a concentration of 2–4 worms per °l and 3mL of worms in S-basal were transferred using sterile technique to capped Pyrex glass culture tubes. Tubes were placed on a rocking platform at 20°C for the duration of the survival.

L1 survival was measured by sampling starved populations using a sterile glass pipette and depositing 50–100 L1 arrested animals (yielding 25–50 extrachromosomal array animals depending on transgene transmission rates) onto each of 2–3 NGM plates seeded with OP50 *E*. *coli* bacteria. Plates were incubated at 20°C for 3 days, after which the number of animals that had progressed passed the third larval stage were expressed as a percentage of total animals loaded. Survival percentages for each technical sampling were averaged together to give the survival percentage for each time point. Animals were sampled three to four times between the first and tenth day of starvation in equal intervals followed by measurements every 2–3 days afterwards until all animals were dead.

### Measurement of M-cell lineage cell divisions

Animals bearing the *hlh-8*::GFP reporter were prepared as stated above for L1 survival. At each timepoint, 200–300 animals were sampled in triplicate per genotype and the number of animals with more than 1 GFP-positive cell was recorded.

### Fluorescent imaging

Images of the *daf-2b*::tdTomato single splicing reporter (MGL371) were taken using a Zeiss AxioCam Icm 1 monochromatic camera on a Zeiss Axio Observer A1 inverted microscope. Images of the dual splicing reporter (MGL456) and the *daf-2b*::*mScarlet* translational fusion (MGL370) were taken using a Zeiss AxioCam MRm camera on a Zeiss Axio Imager M1 microscope. For co-localization imaging the optimal exposure time was selected for each channel to obtain a similar intensity of signal. To quantify the differences in *daf-2b*::tdTomato and *daf-2a/c*::mNeon Green from the dual splicing reporter strain in fed and starved conditions, the exposure time and camera settings were identical in all conditions and in all channels. For representative images shown in Fig 5, the exposure time was increased in the fed condition relative to the starved since the fluorescent signal was low. Average pixel intensity in a given region of interest was calculated from grayscale TIFF images using ImageJ.

### Statistics and replication

The sample size for each experiment was determined empirically and was based on accepted practice within the *C*. *elegans* field. Statistical analysis was performed using GraphPad Prism v 9.0 with p < 0.05 indicating significance. The Log-Rank test was used to analyze L1 starved animals by simulating a survival rate to 100 arbitrary worms [25]. Each technical replicate per time point was averaged and rounded to the nearest whole worm as a percentage of surviving fraction. For transgenic overexpression lines, technical replicates from each of three individual lines were averaged together and compared to control. Student t-test and two-way ANOVA with a post-hoc pairwise test for multiple comparisons were used for comparisons between k = 2 or k>2 groups respectively.

## Results

### Genetic loss of *daf-2b* reduces starvation survival

**Fig 1:**
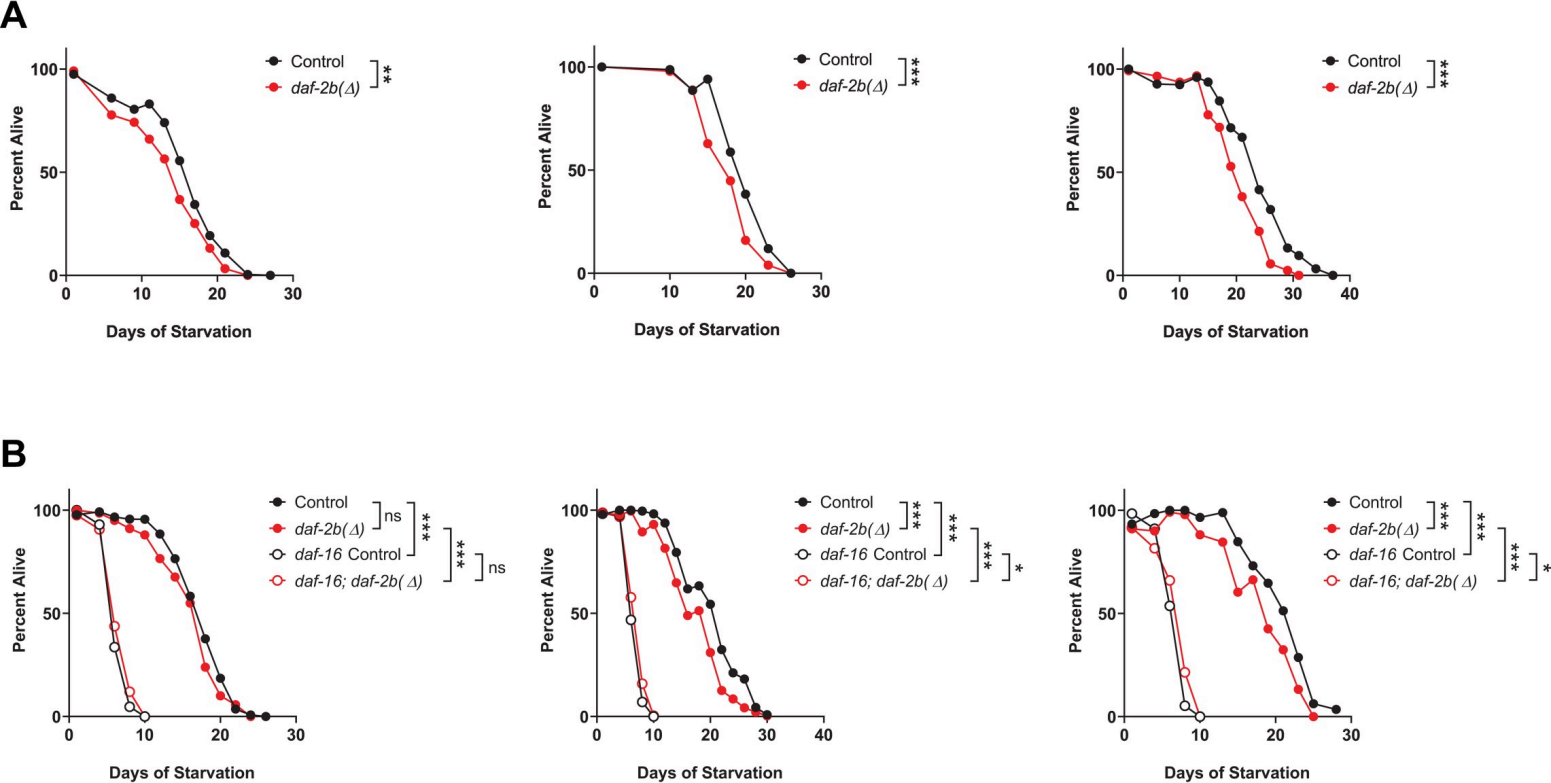
Genetic loss of *daf-2b* reduces survival during L1 starvation. **(A)** L1 starvation survival is reduced in *daf-2b(Δ)* mutants compared with control. **(B)** Genetic deletion of *daf-16* reduces L1 starvation survival in controls and *daf-2b(Δ)* mutants. Each graph represents an independent biological replicate. Log Rank test, *** p<0.001, *p<0.05.

Starvation survival in L1 larvae is increased in *daf-2* hypomorph mutants and decreased in *daf-16* mutants, indicating that reduced insulin signaling promotes L1 starvation survival [11]. We have previously demonstrated that genetic deletion of *daf-2b* can promote insulin signaling under conditions in which ILP agonists prevail and reduce insulin signaling when ILP antagonists predominate [21]. Thus, we hypothesized that genetic deletion of *daf-2b* has the potential to either enhance or suppress L1 starvation survival depending on which ILPs predominate. To this end, we observed that starvation survival in arrested L1 animals was reduced in *daf-2b(Δ)* mutants compared to controls in 3/3 replicate trials (Fig 1A) suggesting that the normal function of DAF-2B is to antagonize insulin signaling during L1 diapause. Given that IIS drives the survival of arrested L1s, we wished to determine how loss of the DAF-16 transcription factor compared with the loss of DAF-2B. In these additional experiments, we confirmed the reduction in survival of *daf-2b(Δ)* mutants compared with control in 2/3 replicates (Fig 1B). Further we confirmed that loss of *daf-16* had a profound effect on the survival of L1 starved animals (Fig 1B). Given the magnitude of the effect of loss of *daf-2b* versus loss of *daf-16* we conclude that DAF-2B plays a minor role in starvation survival. Moreover, the phenotype of the *daf-16; daf-2(Δ)* mutant suggests that the effect of DAF-2B is mediated via activation of DAF-16.

### Genetic loss of *daf-2b* promotes M-lineage cell division

**Fig 2:**
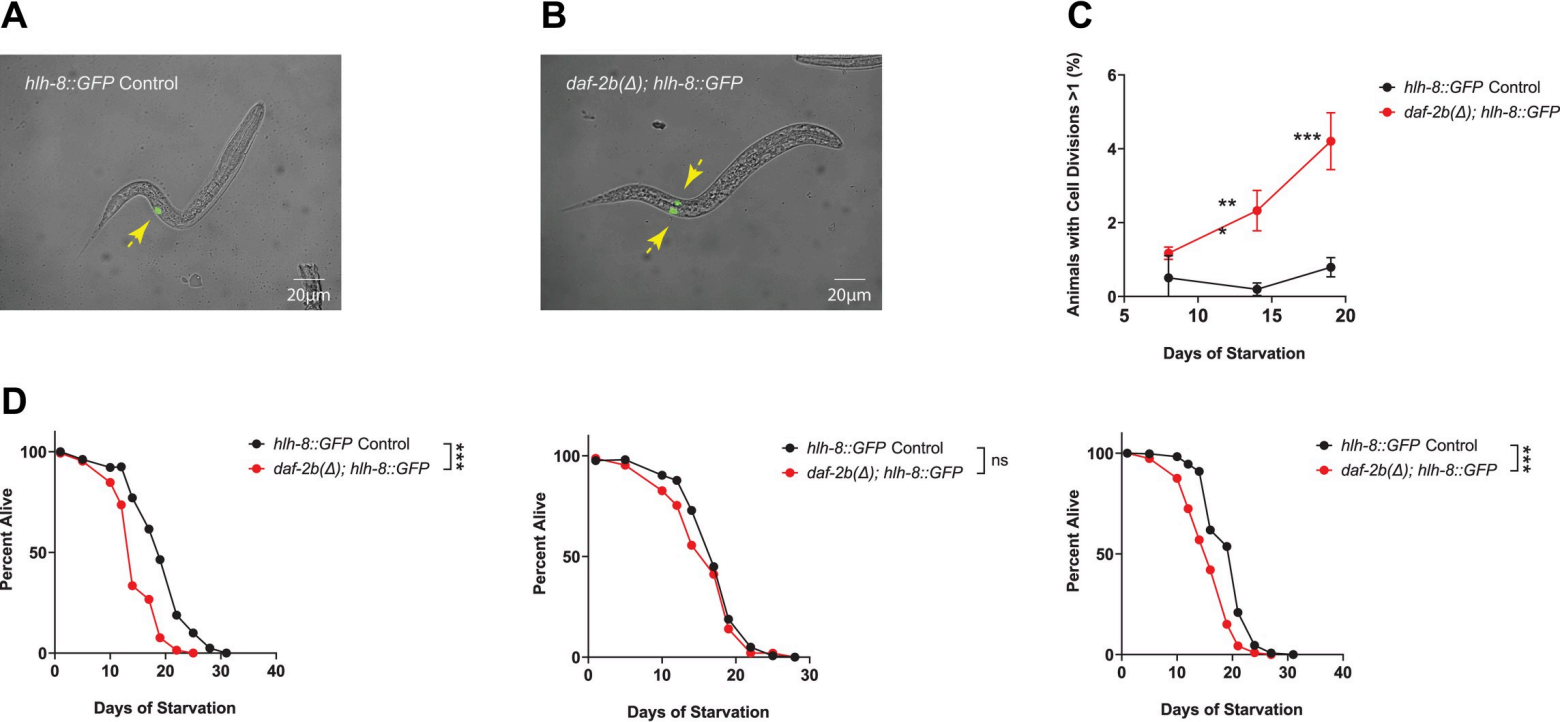
Loss of *daf-2b* increases M-lineage cell divisions. **(A)** Starved L1 animals have a single M-lineage cell marked by *hlh-8*::*GFP* (yellow arrow). **(B)** Starved *daf-2b(Δ)* mutants exhibit increased M-lineage cell divisions (yellow arrows indicate individual cells). **(C)** The percentage of starved animals with more than one M-lineage cell division increases in *daf-2b(Δ)* mutants. Data represent the mean and standard deviation of 3 independent experiments. Student’s *t-*Test, *** *p*<0.001. **(D)** Starvation survival is reduced by *daf-2b* deletion in the *hlh-8*::*GFP* M lineage background. Each graph represents an independent biological replicate. Log Rank test, *** p<0.001.

Reduced IIS during L1 arrest results in cell cycle arrest in several cell lineages, as well as suspending cell migrations [11, 26]. At hatching, L1 animals have a single M-lineage cell that gives rise to 16 cells by the end of the L1 stage under fed conditions, but starved L1s retain only a single cell due to cell division arrest [11]. *daf-16* mutants exhibit an increased frequency of M-lineage cell divisions during L1 arrest indicating that cell cycle quiescence during starvation is compromised if reduced insulin signaling is not maintained [11]. Using the *hlh-8*::*GFP* M-lineage marker, we also found an increased frequency of M-lineage cell divisions in *daf-2b(Δ)* mutants under starvation conditions compared with controls (Fig 2A–2C). Importantly, we also observed decreased starvation survival in the *hlh-8*::*GFP; daf-2b(Δ)* background in 2/3 replicates (Fig 2D) providing additional evidence for the role of DAF-2B in L1 starvation survival in another genetic background.

### Splicing capacity of *daf-2b* is retained in the hypodermis during L1 arrest

**Fig 3:**
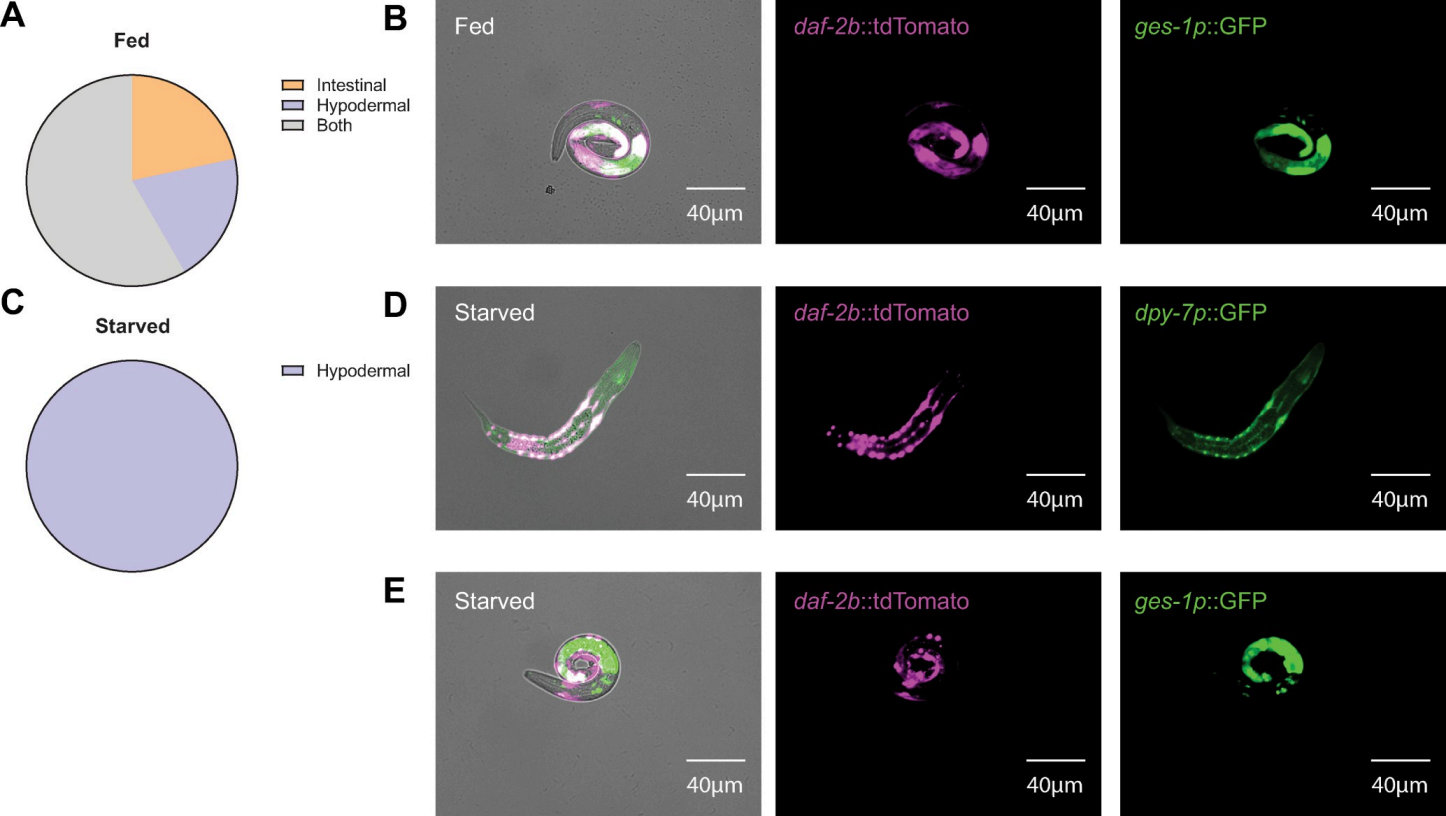
*daf-2b* splicing capacity is predominantly hypodermal during L1 starvation. **(A)** In fed L1 animals *daf-2b* splicing capacity is observed predominantly in the intestine, Data were derived by qualitatively assessing well-fed animals (n = 30) across two independent trials (total n = 60). **(B)** Representative images of well-fed L1 animals showing colocalization between the *daf-2b*::*tdTomato* splicing reporter (exposure time 500ms) and intestinal *ges-1p*::*GFP* (exposure time 300ms). **(C)** In starved L1 animals *daf-2b* splicing capacity is observed mainly in the hypodermis. Data were derived by qualitatively assessing starved animals (n = 30) across two independent trials (total n = 60). **(D)** Representative images of starved L1 animals showing co-localization of the *daf-2b*::tdTomato splicing reporter (exposure time 100ms) and hypodermal *dpy-7p*::*GFP* (exposure time 750ms). **(E)** Representative images illustrating the lack of co-localization between the *daf-2b*::*tdTomato* splicing reporter (exposure time 150ms) and intestinal *ges-1p*::*GFP* (exposure time 75ms). Images were taken at 20x magnification.

Using a *daf-2b*-specific splicing reporter, we have previously observed *daf-2b* splicing capacity in the intestine and hypodermis in fed L1 larvae [21]. To determine if *daf-2b* splicing is changed during L1 starvation, we subjected animals to starvation conditions and compared expression of the splicing reporter to well-fed L1 populations. We repeated our previous observation that the intestine is the major site of *daf-2b* splicing capacity in fed L1 animals (Fig 3A & 3B). In contrast, in starved L1s we found that the *daf-2b* splicing signal was enriched in the hypodermis (Fig 3C and 3D), with very little intestine expression (Fig 3E).

### DAF-2B is a secreted protein in arrested L1 animals

**Fig 4:**
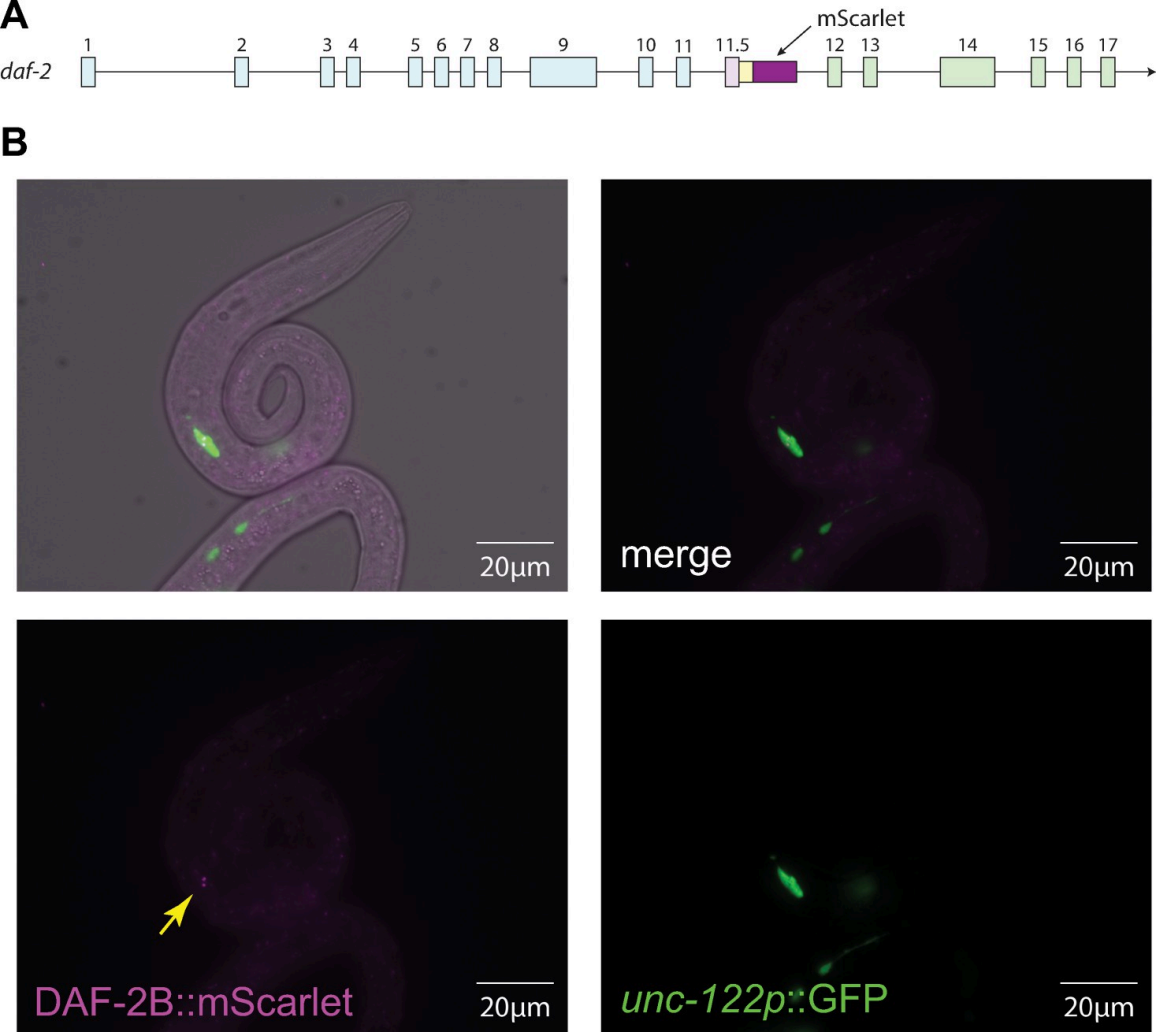
DAF-2B protein is secreted in starved L1 larvae. **(A)** Schematic illustrating the location of mScarlet insertion into the *daf-2* genomic locus. **(B)** Representative image showing localization of DAF-2B::mScarlet protein in coelomocytes marked with *unc-122p*::GFP in starved L1 larvae. Yellow arrow indicates DAF-2B::mScarlet in coelomocyte. Images were taken at 63x magnification.

We have previously generated a DAF-2B translational reporter by using CRISPR/Cas9 gene editing to insert the mScarlet fluorescent protein into the DAF-2 genomic locus (Fig 4A) [21]. Using this line, we previously showed that DAF-2B is a secreted protein, based on the presence of DAF-2B::mScarlet fluorescence in macrophage-like coelomocyte cells in fed L1s, as well as adults [21]. In this study, we confirmed that DAF-2B::mScarlet can also be observed in the coelomocytes of starved L1 animals (Fig 4B). This suggests that during L1 arrest, DAF-2B is a secreted protein and, based on the splicing reporter data, it likely originates from the hypodermis.

### Altered *daf-2b* splicing is elevated compared to full-length isoforms

**Fig 5:**
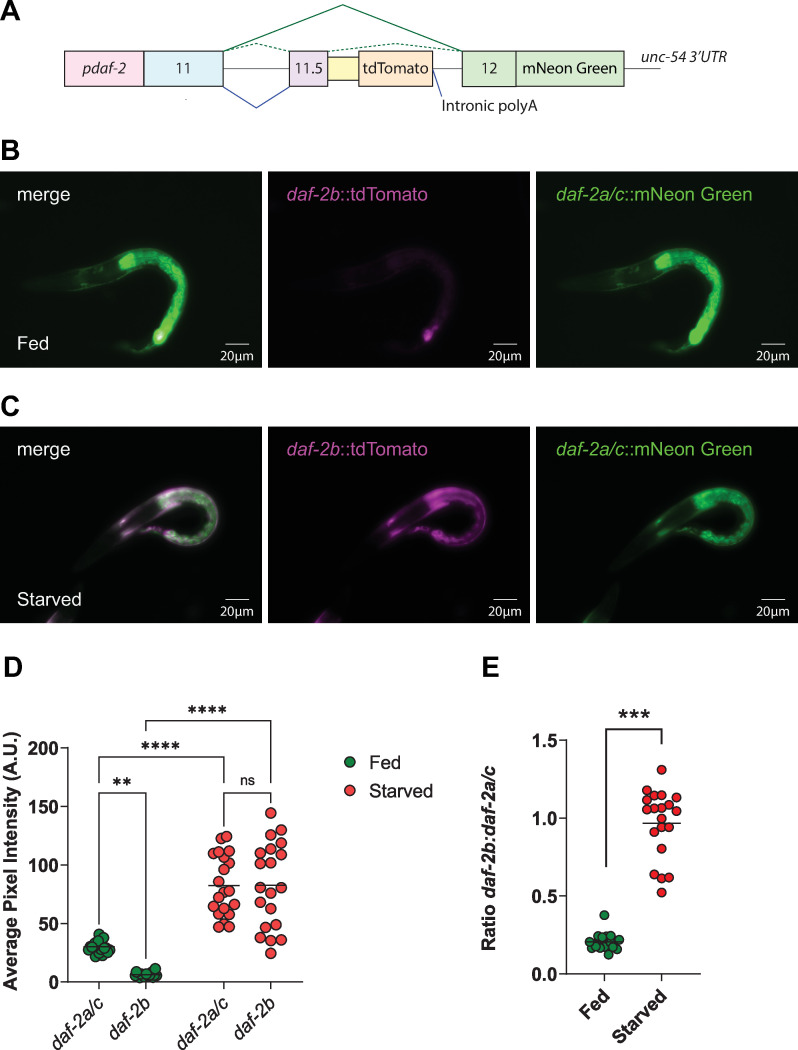
The ratio of *daf-2b*:*daf-2a/c* splicing increases in L1 starvation. **(A)** Schematic illustrating the organization of the *daf-2* dual splicing reporter. mNeon Green fluorescence provides a readout of *daf-2a/c* splicing capacity while tdTomato fluorescence reflects *daf-2b* splicing capacity. **(B)** Representative images of fed L1 larvae expressing the dual reporter. Exposure time for each fluorescence channel - 250ms. **(C)** Representative images of starved L1 larvae expressing the dual reporter. Exposure time for each fluorescence channel 50ms. **(D)** Quantitation of *daf-2b* and *daf-2a/c* splicing capacity in fed and starved L1 animals. Image analysis was performed on images taken with exposure time of 50ms. **(E)** There is a significant increase in the ratio of *daf-2b*:*daf-2a/c* in starved L1 larvae compared with fed L1 animals. Student t-test, ***p<0.001. Images were taken at 40x magnification.

We hypothesized that the extent to which DAF-2B can modify insulin signaling is likely to be dependent on the ratio between the truncated isoform and full-length insulin receptors. In this respect, reduced insulin signaling is more likely to occur if DAF-2B expressions exceeds or is close to the expression of the full-length receptors in any given tissue. We have previously examined *daf-2b* and *daf-2a/c* splicing in independent transgenic lines expressing different tdTomato-based splicing reporters [21]. While this allows for a qualitative comparison of tissue localization, differences in copy number between transgenic lines precludes a comparison of the relative abundance of *daf-2b* versus *daf-2a/c* splicing capacity. To circumvent this, we generated a dual splicing reporter which expresses tdTomato as a function of *daf-2b* splicing and mNeon Green as a function of *daf-2a/c* splicing (Fig 5A). Importantly, both fluorophores are encoded by the same expression construct, allowing comparison of the relative abundance of each splicing event. These reporters give a read out of splicing capacity, which is a measure of the ability of specific tissues to generate fluorescent protein expression in a manner that is consistent with the splicing of endogenous *daf-2b*, *daf-2a*, *and daf-2c* transcripts.

In fed L1s, the tissue distribution of *daf-2a/c* splicing capacity was predominantly intestinal with some hypodermal and neuronal signal (Fig 5B). The tissue distribution of *daf-2b* splicing capacity under these conditions was similar, but with a much lower signal (Fig 5B), consistent with the single *daf-2b* splicing reporter [21]. During L1 starvation, there was strong co-localization of *daf-2b* and *daf-2a/c* signal in the hypodermis (Fig 5C). Interestingly, the *daf-2a/c* splicing signal was still present in the intestine, a tissue where *daf-2b* was absent, suggesting that this tissue remains insulin responsive. Using images taken at the same exposure for all conditions, we then quantified each splicing signal in the two conditions. In fed L1s, the splicing of the full-length *daf-2a/c* reporter was significantly greater than that of *daf-2b* (Fig 5D). We interpret this as an insulin responsive state, which would be expected given the role of insulin signaling in promoting reproductive growth. However, there was a significant increase in the levels of each splicing reporter in starved L1 animals compared to fed L1 animals (Fig 5D). Moreover, there was a dramatic change in the ratio of the two splicing signals in starved animals. Under fed conditions the ratio of *daf-2b*:*daf-2ac* was around 0.2, indicating a greater abundance of *daf-2a/c* splicing compared to *daf-2b* (Fig 5E). In contrast, in starved animals, this ratio increased to almost 1, indicating that the level of *daf-2b* splicing was similar to that of *daf-2a/c* (Fig 5E). If this splicing ratio were reflected in endogenous transcript levels it would suggest that a greater ability of DAF-2B to sequester insulin peptides therefore leading to reduced insulin responsiveness in those tissues.

### Overexpression of DAF-2B in the hypodermis improves starvation survival in L1 larvae

**Fig 6:**
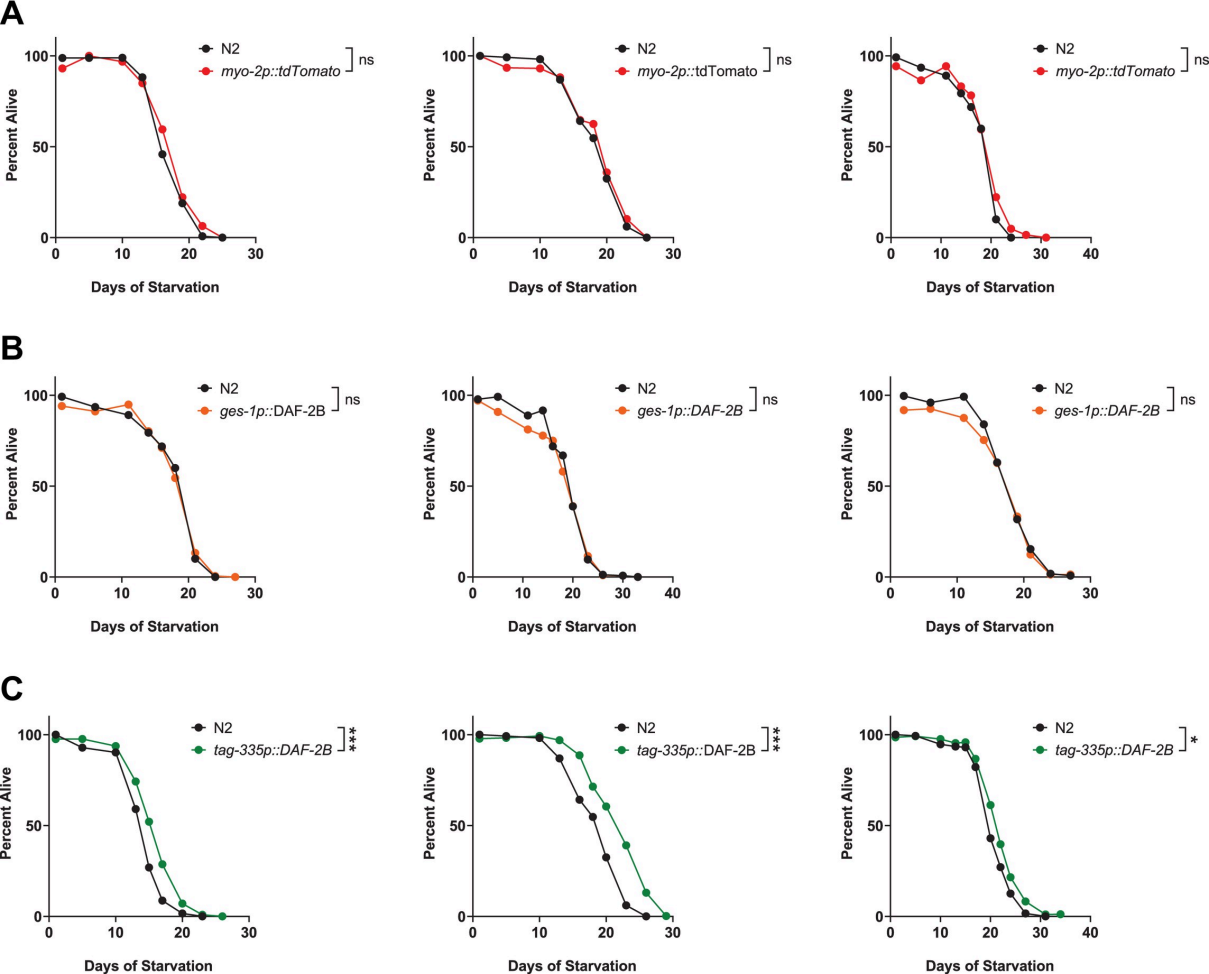
Overexpression of *DAF-2B* in the hypodermis improves survival of L1 starved animals. **(A)** The *myo-2p*::*tdTomato* transgene has no effect on L1 starvation survival. **(B)** Expression of *daf-2b* cDNA from the intestinal *ges-1* promoter has no effect on L1 starvation survival. **(C)** Expression of *daf-2b* cDNA from the hypodermal *tag-335* promoter increases L1 starvation survival. Log Rank test, *** p < .0001. Data compiled from three independent transgenic lines except for the marker transgene control which used one. Each graph represents an independent biological replicate.

The switch in DAF-2B splicing capacity from the intestine in fed L1 animals to the hypodermis in starved L1s suggests that this latter tissue is important for maintaining low insulin signaling and promoting L1 survival. To investigate this, we overexpressed DAF-2B from extrachromosomal arrays in the intestine and the hypodermis. When we expressed an injection control (*myo-2p*::*tdTomato*; Fig 6A) or DAF-2B in the intestine (*ges-1p*::*DAF-2B*; Fig 6B) we did not observe any change in L1 starvation survival (3/3 trials for each). In contrast, when we overexpessed DAF-2B in the hypodermis from the *tag-3*35 promoter we observed an increase in starvation survival compared to control animals in 3/3 trials (Fig 6C). This suggests that DAF-2B expression in the hypodermis is sufficient to increase starvation survival in L1 larvae.

## Discussion

Reduced insulin signaling is an important feature of starvation survival in L1 larvae [1]. Under fed conditions, insulin-like peptides bind to the nematode insulin receptor DAF-2, activating an intracellular kinase pathway that inactivates DAF-16/ FOXO transcription factor via phosphorylation [9]. Conversely, under starved conditions reduced insulin signaling leads to activation of DAF-16 which in turn promotes survival [11].

We have previously observed that the truncated DAF-2B isoform acts as a modifier of insulin signaling in the context of the dauer diapause by altering the response to endogenously secreted insulin-like peptides [21]. In this study, we discovered that genetic deletion of *daf-2b* resulted in a reduction in L1 survival. This suggests that in the absence of DAF-2B, the animals have higher insulin signaling, therefore supporting a role for DAF-2B in attenuating the activity of agonist insulin peptides. In contrast to the large decrease in survival conferred by *daf-16* mutation, the effect of *daf-2b* deletion was much more modest and consistent with a role for DAF-2B as a modifier of L1 arrest.

Many of the genes that are involved in L1 arrest, such *aak-2* / AMPK [2], *skn-1* / NRF-2 [27], *daf-2* / INSR [11], play large, indispensable roles in the signal transduction pathways in which they operate. However, to drive physiological change animals also use molecular modifiers that aid and reinforce broader biological processes through combinatorial interactions. For instance, microRNAs which regulate gene expression have been implicated in regulating transcriptional activity during L1 starvation [25]. The total combined efforts of modifiers likely provide plasticity to animals to help in adapting to constantly changing, uncertain environments and as such, identification of potential modifiers and their activities is important to understand the complexity of stress responses.

In other systems, binding proteins have been described that act as modifiers of insulin and ILP signaling [28]. In mammals, the circulating half-life and bioactivity of insulin-like growth factor-I (IGF-I) and IGF-II are regulated by a set of IGF binding proteins (IGFBPs) [29]. Similarly, in *Drosophila* where there are 6 ILPs, binding proteins for insulin-like peptides have been reported [30, 31]. The existence of similar binding proteins in *C*. *elegans* would provide an attractive mechanism by which activity of different ILPs could be regulated. The fact that DAF-2B is secreted and acts in an ILP-dependent manner is consistent with the action of an ILP-binding protein. However, given the number and variety of ILPs in worms, it seems likely that other ILP binding proteins could exist. Intriguingly, a family of genes that encode proteins with homology to the extracellular ligand binding domain of the insulin and EGF receptors (*irld* genes) [32] have been recently been implicated in L1 starvation resistance in wild derived strains of *C*. *elegans* [33]. Moreover, genetic deletion of two of these genes was sufficient to extend L1 starvation survival [33]. Thus, it is tempting to speculate that these two IRLD proteins are acting to sequester antagonist ILPs and could form part of a larger family of ILP binding proteins.

Using a *daf-2b*-specific splicing reporter, we found *daf-2b* splicing activity in starved L1s was enriched in the hypodermis, while a single copy translational fusion suggested that DAF-2B remains a secreted protein. In contrast, in fed animals *daf-2b* splicing fluorescence was observed predominantly in the intestine. The nervous system, the intestine and the hypodermis have all been shown to be important for maintaining low insulin signaling and promoting starvation survival [3]. Many ILPs are neuronal in origin and control of their secretion by UNC-31/CAPS in a subset of neurons has been shown to be important for L1 arrest [34]. However, other ILPs are secreted from the intestine via mechanism that involves the ATPase ASNA-1 [35]. Reduced insulin signaling in the hypodermis is important for maintaining cellular quiescence in P and M blast cells [26]. We observed increased cell division in the M lineage in *daf-2b(Δ)* mutants and increased starvation survival when DAF-2B was overexpressed specifically in the hypodermis, suggesting that the primary role of DAF-2B is to promote and / or maintain reduced insulin signaling in this tissue, perhaps by sequestering ILPs from neurons and the intestine.

In addition to examining the changes in ILP expression in fed and starved L1s, Chen and Baugh also observed that *daf-2* gene expression was upregulated in starved animals [20]. This is consistent with our observation that *daf-2a/c* splicing capacity increases in starved L1 animals. However, this increase in *a/c* splicing is set against a similar rise in *daf-2b* splicing capacity, such that the whole-body ratio of *daf-2b* to *daf-2a/c* is around one. If these splicing signals were reflected in protein expression the net outcome would be lowered organismal insulin signaling. Interestingly, the colocalization between *daf-2b* and *daf-2a/c* splicing capacity is only observed in the hypodermis, with the intestine exhibiting *daf-2a/c* expression but no *daf-2b* signal. This in turn would suggest that the intestine retains the capacity to be insulin sensitive and is consistent with the proposal that there remains a basal level of insulin signaling during L1 arrest [20]. This hypothesis comes from the observations that loss of insulin peptide release in *unc-31* mutants [34], loss of insulin responsiveness in *daf-2* mutants [11], as well as the specific loss of agonist insulin peptides *daf-28* and *ins-4* [20] all result in increased starvation survival. Our observations with *daf-2b* deletion and overexpression lend further support to this idea.

The ability of an animal to respond quickly upon refeeding is perhaps as important, if not more important, than the ability to survive starvation itself. This has been elegantly demonstrated with *C*. *elegans* insulin signaling mutants in competition experiments [36]. *age-1* / PI3K mutants have reduced insulin signaling and are consequently stress resistant and long-lived [37, 38]. Under nutrient replete conditions, there is no fitness cost associated with this mutation and the *age-1* allele can compete favorably with N2 animals [36]. However, under cycles of starvation, the *age-1* allele is out-competed by N2. This demonstrates there is a fitness cost in reduced IIS mutants like *age-1* following starvation such that they do not resume reproduction as quickly as wild type animals [36]. In the context of L1 diapause, a tradeoff between maximally reducing insulin signaling to promote starvation survival and leaving enough residual insulin signaling to facilitate rapid recovery upon refeeding may exist. In this respect, DAF-2B may play an important role in maintaining a low insulin signaling state in the hypodermis, whilst leaving the intestine insulin-sensitive.

## Supporting information

S1 File List of primers (S1 Table), plasmids (S2 Table) and strains (S3 Table) used in this study. (DOCX)

S2 File All raw survival data for Figs 1, 2 and 6. (XLSX)
