# Supplementary material for: The C. elegans truncated insulin receptor DAF-2B regulates survival of L1 arrested larvae
Source: PLoS One. 2023 Jul 20;18(7):e0288764. doi: 10.1371/journal.pone.0288764 (PMC10358897; doi:10.1371/journal.pone.0288764)
Supplement: S1 File — (DOCX) [file pone.0288764.s001.docx]

## Supporting Information

**Table S1: Primers**

**Table S2: Plasmids**

**Table S3: Strains**

## Table S1: Primers

| **Purpose** | **Primers** |
| --- | --- |
| *daf-2* dual splicing reporter | 1 CCGGTACCGGTAATGGTGTCGAAGGGAGAAGAGGATAACATGGC |
|  | 2 CAGCGGCCGATGCGGAGCTCCTACTTGTAGAGTTCATCCATTCCC |
|  | 3 GGGAATGGATGAACTCTACAAGTAGGAGCTCCGCATCGGCCGCTG |
|  | 4 CGTACGGCCGACTAGTAGGAAACAGTTA |
|  | 5 CCGCTCGAGGCGCGAAATTTGAATTTTTCAAAAAAAATTCG |
| *daf-2bc(Δ)* genotyping | 6 TGCATTTGAGAATAAGCTGTTGG |
|  | 7 TATGCCTGCTCCAAGCCTAT |
|  | 8 CGCATAAAGTGTGTAATGCTTCAAAT |
| MosSCI genotyping | 9 CGCTACTTACCGGAAACCAA |
|  | 10 TTTCTCAGTTGTGATACGGTTTTT |
|  | 11 CAATTCATCCCGGTTTCTGT |
|  | 12 TCTGGCTCTGCTTCTTCGTT |
| *daf-16(mu86)* genotyping | 13 ACTGTCTACCTCTCCTCCTG |
|  | 14 CAGAATAGGTGTGCGCCT |
|  | 15 CGACTCCTGCTTAATCTGAACTC |

## Table S2: Plasmids

| **Name** | **Description** | **Comments** |
| --- | --- | --- |
| dg356 | (pENTRslot2_NeonGreen_NLS_stop) | [1] |
| pRF4 | *rol-6(su1006)* | Co-injection marker |
|  | *myo-2p::tdTomato* | Co-injection marker. Gift from Will Mair |
| pMGL83 | pBGY487 *pdaf-2::daf-2a/c::*tdTomato minigene | *daf-2a/c* splicing reporter [2] |
| pMGL86 | pBGY487 *pdaf-2::daf-2b::*tdTomato minigene | *daf-2b* splicing reporter [2] |
| pMGL123 | pPD49.26 *pges-1::daf-2b* cDNA | Intestinal *daf-2b* expression [2] |
| pMGL150 | pPD49.26 *ptag-335::daf-2b* cDNA | Hypodermal *daf-2b* expression [2] |
| pMGL116 | pPD49.26 *pdpy-7::GFP* | Hypodermal GFP expression [2] |
| pMGL117 | pPD49.26 *pges-1::GFP* | Intestinal GFP expression [2] |
| pMGL225 | pBGY487 *pdaf-2::daf-2a/c::mNeon Green* minigene | *daf-2a/c* splicing reporter. This study |
| pMGL226 | pBGY487 *pdaf-2::daf-2b::tdTomato daf-2a/c::mNeon Green* minigene | *Daf-2b::tdTomato daf-2a/c::mNeon Green* dual splicing reporter. This study. |

## Table S3: Strains

| **Strain** | **Genotype** | **Source** | **Description** |
| --- | --- | --- | --- |
| N2 | Wild type | CGC | N2 Bristol |
| PD4667 | *ayIs7[hlh-8::GFP fusion + dpy-20(+)]* | CGC | M-lineage reporter |
| CF1038 | *daf-16(mu86)* | CGC | *daf-16* deletion |
| MGL264 | *jluIs15[daf-2p::DAF-2bexon-11.5::tdTomato + rol-6(+)]* | [2] | Integrated *daf-2b* splicing reporter |
| MGL297 | *jluSi3[daf-2p::DAF-2C + unc-119(+)]* | [2] | *daf-2b(Δ)* control strain |
| MGL302 | *jluSi3[daf-2p::DAF-2C + unc-119(+)]; daf-2(jlu1)* | [2] | *daf-2b(Δ)* |
| MGL364 | *jluIs15; jluEx180[dpy-7p::GFP]* | [2] | *daf-2b* splicing reporter + hypodermal GFP |
| MGL365 | *jluIs15; jluEx181[ges-1p::GFP]* | [2] | *daf-2b* splicing reporter + intestinal GFP |
| MGL370 | *daf-2(jlu2[daf-2b::mScarlet]); jluEx184[unc-122p::GFP]* | [2] | *daf-2b::mScarlet* CRISPR Knock-in + coelomocyte GFP expression |
| MGL371 | *jluIs15[daf-2p::DAF-2bexon-11.5::tdTomato + rol-6(+)]* | *This study* | Integrated *daf-2b* splicing reporter |
| MGL476 | *daf-16(mu86)* | *This study* | 3X outcross |
| MGL477 | *daf-16(mu86); jluSi3[daf-2p::DAF-2C + unc-119(+)]* | *This study* | *daf-16* deletion in *daf-2b(Δ)* control background |
| MGL478 | *daf-16(mu86); jluSi3[daf-2p::DAF-2C + unc-119(+)]; daf-2(jlu1)* | *This study* | *daf-16* deletion in *daf-2b(Δ)* deletion background |
| MGL474 | *jluSi3[daf-2p::DAF-2C + unc-119(+)]; ayIs7[hlh-8::GFP fusion + dpy-20(+)]* | *This study* | M-lineage GFP reporter in *daf-2b(Δ)* control background |
| MGL475 | *jluSi3[daf-2p::DAF-2C + unc-119(+)]; daf-2(jlu1); ayIs7[hlh-8::GFP fusion + dpy-20(+)]* | *This study* | M-lineage GFP reporter in *daf-2b(Δ)* deletion background |
| MGL456 | *jluIs19[daf-2p::DAF-2bexon-11.5::tdTomato:: DAF-2a/cexon-12::mNeon Green + rol-6(+)]* | *This study* | *daf-2b::tdTomato – daf-2a/c::mNeon Green* dual splicing reporter |
| MGL465  MGL466  MGL467 | *jluEx198[tag-335p::DAF-2B + myo-2p::tdTomato]*  *jluEx199[tag-335p::DAF-2B + myo-2p::tdTomato]*  *jluEx200[tag-335p::DAF-2B + myo-2p::tdTomato]* | *This study* | Hypodermal DAF-2B overexpresser |
| MGL468  MGL469  MGL470 | *jluEx201[ges-1p::DAF-2B + myo-2p::tdTomato]*  *jluEx202[ges-1p::DAF-2B + myo-2p::tdTomato]*  *jluEx203[ges-1p::DAF-2B + myo-2p::tdTomato]* | *This study* | Intestinal DAF-2B overexpresser |
| MGL471 | *jluEx204[myo-2p::tdTomato]* | *This study* | *myo-2p::*tdTomato control line |

**References**

1. Hostettler L, Grundy L, Kaser-Pebernard S, Wicky C, Schafer WR, Glauser DA. The Bright Fluorescent Protein mNeonGreen Facilitates Protein Expression Analysis In Vivo. G3. 2017;7(2):607-15. Epub 2017/01/22. doi: 10.1534/g3.116.038133. PubMed PMID: 28108553; PubMed Central PMCID: PMCPMC5295605.

2. Martinez BA, Reis Rodrigues P, Nunez Medina RM, Mondal P, Harrison NJ, Lone MA, et al. An alternatively spliced, non-signaling insulin receptor modulates insulin sensitivity via insulin peptide sequestration in C. elegans. eLife. 2020;9. Epub 2020/02/26. doi: 10.7554/eLife.49917. PubMed PMID: 32096469; PubMed Central PMCID: PMCPMC7041946.
